# Adaptive resistance to therapeutic PD-1 blockade is associated with upregulation of alternative immune checkpoints

**DOI:** 10.1038/ncomms10501

**Published:** 2016-02-17

**Authors:** Shohei Koyama, Esra A. Akbay, Yvonne Y. Li, Grit S. Herter-Sprie, Kevin A. Buczkowski, William G. Richards, Leena Gandhi, Amanda J. Redig, Scott J. Rodig, Hajime Asahina, Robert E. Jones, Meghana M. Kulkarni, Mari Kuraguchi, Sangeetha Palakurthi, Peter E. Fecci, Bruce E. Johnson, Pasi A. Janne, Jeffrey A. Engelman, Sidharta P. Gangadharan, Daniel B. Costa, Gordon J. Freeman, Raphael Bueno, F. Stephen Hodi, Glenn Dranoff, Kwok-Kin Wong, Peter S. Hammerman

**Affiliations:** 1 Department of Medical Oncology and Cancer Vaccine Center, Dana Farber Cancer Institute, Boston, Massachusetts 02215, USA; 2 Depatment of Medicine, Brigham and Women's Hospital and Harvard Medical School, Boston, Massachusetts 02115, USA; 3 Department of Medical Oncology, Dana Farber Cancer Institute, Boston, Massachusetts 02215, USA; 4 Department of Thoracic Surgery, Brigham and Women's Hospital, Boston, Massachusetts 02115, USA; 5 Department of Pathology, Brigham and Women's Hospital, Boston, Massachusetts 02115, USA; 6 Belfer Institute for Applied Cancer Science, Dana Farber Cancer Institute, Boston, Massachusetts 02215, USA; 7 Division of Neurosurgery, Department of Surgery, Duke University Medical Center, Durham, North Carolina 27710, USA; 8 Massachusetts General Hospital Cancer Center, Boston, Massachusetts 02114, USA; 9 Beth Israel Deaconess Medical Center, Boston, Massachusetts 02215, USA; 10 Cancer Program, Broad Institute of Harvard and MIT, Cambridge, Massachusetts 02142, USA

## Abstract

Despite compelling antitumour activity of antibodies targeting the programmed death 1 (PD-1): programmed death ligand 1 (PD-L1) immune checkpoint in lung cancer, resistance to these therapies has increasingly been observed. In this study, to elucidate mechanisms of adaptive resistance, we analyse the tumour immune microenvironment in the context of anti-PD-1 therapy in two fully immunocompetent mouse models of lung adenocarcinoma. In tumours progressing following response to anti-PD-1 therapy, we observe upregulation of alternative immune checkpoints, notably T-cell immunoglobulin mucin-3 (TIM-3), in PD-1 antibody bound T cells and demonstrate a survival advantage with addition of a TIM-3 blocking antibody following failure of PD-1 blockade. Two patients who developed adaptive resistance to anti-PD-1 treatment also show a similar TIM-3 upregulation in blocking antibody-bound T cells at treatment failure. These data suggest that upregulation of TIM-3 and other immune checkpoints may be targetable biomarkers associated with adaptive resistance to PD-1 blockade.

## Abstract (web summary)

Blocking immune checkpoints is a promising strategy to treat lung cancer, but patients often become resistant to the therapy. Here, the authors analyse resistance in mouse models of lung cancer and show in mice and two patients, an increase in the expression of TIM3, which is also involved in the immune response to cancer.

## Graphical abstract

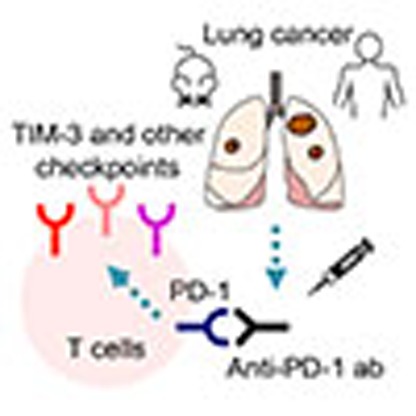

Programmed death 1 (PD-1): Programmed death ligand 1 (PD-L1) immune checkpoint blockade has been demonstrated to be efficacious in a number of cancer types, including melanoma, renal cell carcinoma, bladder cancer, hematologic malignancies and non-small cell lung cancer (NSCLC)^1^,^2^,^3^ and anti-PD-1 antibodies have recently been approved for use in the United States and Asia. Anti-PD-1 therapeutic antibodies function through binding to PD-1 on tumour-reactive T cells and inhibiting the PD-1:PD-L1 interaction, thereby reinvigourating the anti-tumour T-cell response^4^,^5^,^6^. Expression of PD-L1 in tumour cells and infiltrating immune cells and PD-1 in tumour-infiltrating T cells has been associated with responsiveness to blockade of this immune checkpoint^1^,^7^,^8^,^9^,^10^; however, mechanisms of both *de novo* and adaptive resistance to therapy are unclear.

NSCLC is the leading cause of cancer-related mortality world-wide. While its treatment has been dramatically improved in patients who harbour targetable genomic alterations including epidermal growth factor receptor (*EGFR*) mutations and anaplastic lymphoma kinase (*ALK*) fusions^11^, it remains the case that only a minority of NSCLC patients benefit from these targeted agents. Conversely, immunotherapy approaches, specifically PD-1:PD-L1 blockade, appear to be broadly efficacious in NSCLC patients^12^,^13^,^14^. Although the mechanisms of resistance to targeted kinase inhibitors have been extensively studied^15^,^16^, there have been no studies reported to date of resistance to PD-1:PD-L1 blockade. We previously reported that a fully immunocompetent mouse model of lung cancer driven by expression of mutated *EGFR* demonstrates responsiveness to PD-1 blockade associated with augmentation of an anti-tumour T-cell response^17^.

Here we have extended these studies using two genetically engineered mouse models of lung adenocarcinomas corresponding to the two most common oncogene drivers in human lung adenocarcinoma, Kirsten rat sarcoma viral oncogene homologue (*KRAS*) and *EGFR*. The EGFR and Kras models were treated with a therapeutic anti-PD-1 antibody until tumours demonstrated progression by magnetic resonance imaging (MRI) and evaluated immune profiles. We identified that upregulation of other immune checkpoints, most notably TIM-3, on therapeutic antibody-bound T cells as a marker of treatment resistance. To determine whether blockade of TIM-3 at the time of resistance might be therapeutically efficacious, we performed TIM-3-blocking treatment in these mice and demonstrated a clinical benefit. To extend these results and determine their applicability to patients treated with anti-PD-1 antibodies, we analysed specimens from two patients who showed an initial response to PD-1 blockade but ultimately developed progressive disease. These cases exhibited similar upregulation of TIM-3 on therapeutic antibody-bound tumour-infiltrating lymphocytes (TILs). These results suggest that targeting alternate immune checkpoints upregulated in the context of PD-1 therapy may extend the benefit of PD-1 blockade in responsive tumours.

## Results

### Checkpoint expression in TILs at resistance to PD-1 blockade

**Figure 1:**
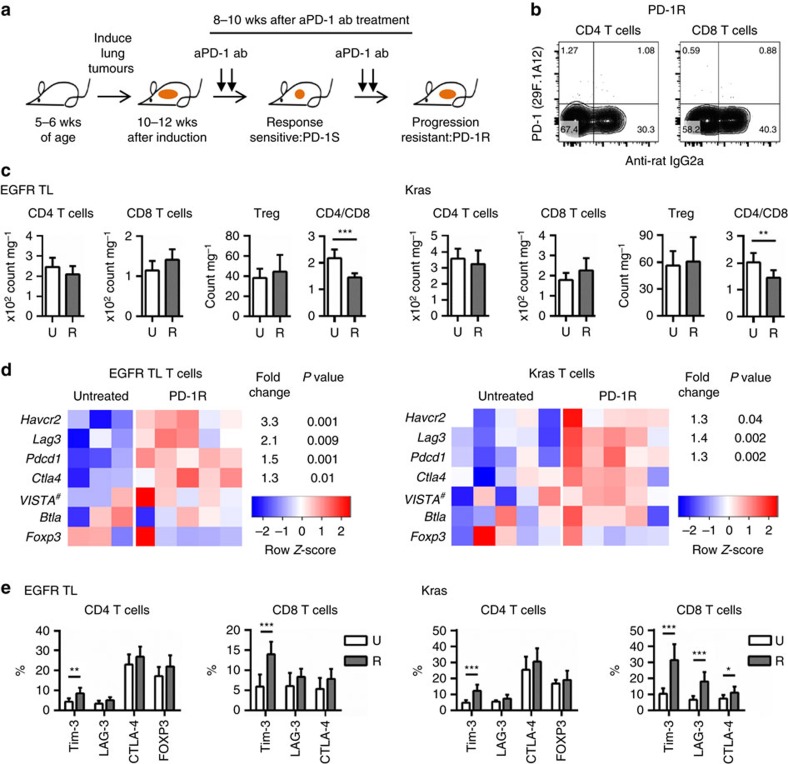
Upregulation of TIM-3 in T cells at the time of acquired resistance to anti-PD-1 blockade. (**a**) Schematic of *in vivo* treatment with anti-PD-1 antibody until adaptive resistance (**b**) Representative flow cytometry data from anti-PD-1 resistant (PD-1R) EGFR TL mouse. PD-1 expression and anti-Rat IgG2a (therapeutic antibody binding) were evaluated. Fluorescent conjugated anti-PD-1 antibody is the same clone (29F.1A12) as the therapeutic antibody. (**c**) Cell number of T cell subsets: CD4 T cells, CD8 T cells and regulatory T cells (Treg) and CD4/CD8 ratio. Untreated (U) EGFR TL (*n*=7), Kras (*n*=7) and anti-PD-1 resistant (R) EGFR TL (*n*=9), Kras (*n*=9) were analysed (EGFR TL ***P<0.001, Kras **P=0.0028, student's *t*-test). Data are shown as mean±s.d. (**d**) Expression of six genes with an annotated role in the T-cell response in sorted T cells from EGFR models (five anti-PD-1 treated and three untreated tumours) in EGFR models and KRAS models (five anti-PD-1 treated and five untreated tumours). For each gene, expression values across the samples are plotted as log-transformed FPKM values (row-scaled and coloured on a blue-red scale to emphasize the difference between treated and untreated samples). The magnitude of change between resistant and genotype-matched untreated samples are shown as fold change and *P* values for differentially expressed genes (defined as having an absolute fold change greater than 1.25 and a *P* value <0.05 as calculated by the limma package^37^). Note the gene name of VISTA is *4632428N05Rik (*Gene ID: 74048). (**e**) Surface expression of inhibitory T cell markers: TIM-3, LAG-3, CTLA-4 and FOXP3. Untreated (U) EGFR TL (*n*=7), Kras (*n*=7) and anti-PD-1 resistant (R) EGFR TL (*n*=9), Kras (*n*=9) were analysed (*P<0.05, **P<0.01, ***P<0.001). Data are shown as mean±s.d.

We performed a therapeutic study of a PD-1 blocking antibody in two fully immunocompetent genetically engineered mouse models of lung cancer, EGFR^T790M/L858R^ (TL) and Kras^G12D^ using the same dosing schedule as described previously^17^ (Fig. 1a). We first compared the lung immune cell populations among untreated (U) and anti-PD-1 treatment-resistant tumours (PD-1R:R) in mice with similar degrees of tumour burden (Supplementary Fig. 1a). Resistance was defined as tumours displaying an initial therapeutic response by MRI imaging (regression or stable disease) followed by growth to >120% of the original tumour size in accordance with the response evaluation in solid tumours (RECIST) definition (Fig. 1a). We also confirmed binding of PD-1-blocking antibody at the time of progression (PD-1R) to exclude decreased antibody binding over time as responsible for the loss of efficacy of PD-1 blockade (Fig. 1b). We found a modest difference in total numbers of CD4 and CD8 T cells, resulting in significantly lower CD4/CD8 ratios in both the EGFR TL and Kras models in resistant nodules (Fig. 1c). There were no significant differences among untreated and anti-PD-1 therapy-resistant samples in forkhead box P3 (FOXP3)^+^ CD4^+^ T cells (regulatory T cells: Treg; Fig. 1c) and two major populations of myeloid cells in the murine models, tumour-associated alveolar macrophages (TAM: CD11c^+^CD11b^−^CD103^−^) and tumour-associated neutrophils (TAN: CD11b^+^Ly6G^+^) (Supplementary Fig. 1b). There were also no significant differences in PD-L1 expression levels in TAM and tumour cells (CD45^−^EpCAM^+^) and levels of the cytokines IL-6 in bronchoalveolar lavage fluids (BALFs), that can promote tumour growth^18^,^19^, between untreated (U) and PD-1 treatment-resistant mice (R) (Supplementary Fig. 1c,d) though the level of IL-6 in BALFs showed a clear reduction in PD-1 treatment-sensitive animals (S) as we previously demonstrated in a different EGFR mutated model^17^.

We next performed a global analysis of sorted T cells and tumour cells by mRNA sequencing of anti-PD-1-resistant versus untreated tumours. In a supervised analysis of genes suppressing T-cell function, we observed in the TL and Kras tumours an increase in the expression of Hepatitis A virus cellular receptor 2 (*Havcr2*, known as *Tim3*), lymphocyte-activation gene 3 (*Lag3*) and programmed cell death 1 (*Pdcd1*; Fig. 1d) but no increase in *Foxp3*, *4632428N05Rik* coding V-domain Ig suppressor of T cell activation: *VISTA*, or *B* and T lymphocyte attenuator (*Btla*). In agreement with the immune profiling results, there was no noticeable expression change in the *Foxp3* expression between treated and untreated tumours. To confirm the expression of these genes at the protein level, we analysed these T-cell inhibitory markers in CD4 and CD8 T cells with flow cytometry analysis. In accordance with the findings from the mRNA sequencing data, TIM-3, LAG-3 and CTLA-4 were expressed at higher levels in both CD4 and CD8 T cells from PD-1 resistant as compared with untreated EGFR TL tumours by flow cytometry analysis. However, only TIM-3 showed a significant increase (Fig. 1e). A significant increase of TIM-3 was also identified in both CD4 and CD8 T cells in the Kras model (Fig. 1e). In addition, there were significant increases in LAG-3 and CTLA-4 expression in CD8 T cells only in Kras tumours, though the magnitude of induction was less than that observed for TIM-3 (Fig. 1e). For PD-1, we found an increasing trend in the percentage of anti-PD-1 antibody bound cells with longer treatment duration when comparing nodules obtained from EGFR TL and Kras mice that had received from 2–8 weeks of therapy (Supplementary Fig. 1e), suggesting that PD-1 blockade could enrich for PD-1 expression on TILs.

### TIM-3 upregulation is time dependent in TILs expressing PD-1

**Figure 2:**
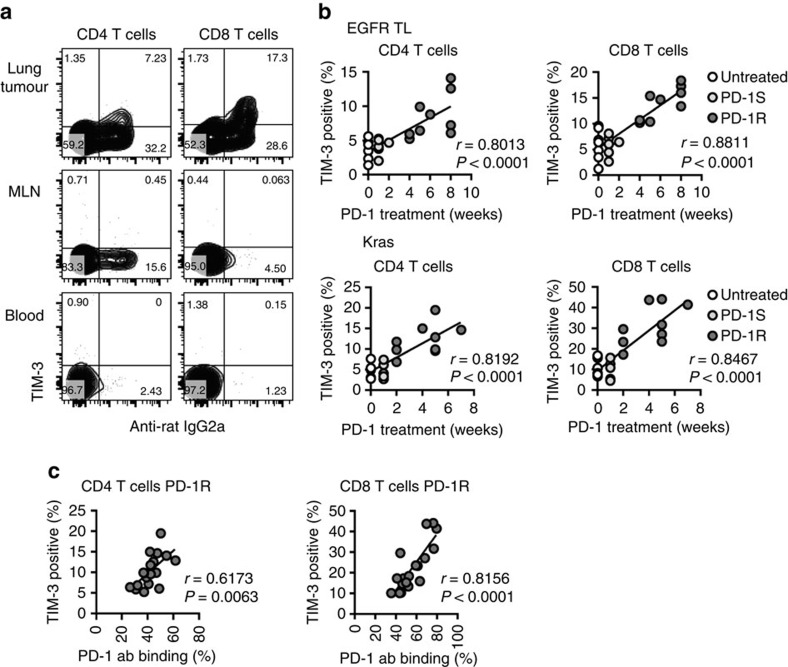
TIM-3 expression in tumour-infiltrating T cells correlates with treatment time and PD-1 antibody binding. (**a**) TIM-3 expression in T cells from tumour-bearing lung, mediastinal lymph node and peripheral blood. Representative flow cytometry data from anti-PD-1 resistant EGFR TL mouse. (**b**) Significant correlation was detected between TIM-3 positivity and the duration of PD-1 blocking treatment in EGFR TL mice (untreated (0 week): *n*=7, anti-PD-1 sensitive (PD-1S): *n*=6 and resistant (PD-1R): *n*=9) and Kras mice (untreated: *n*=7, anti-PD-1 sensitive (PD-1S): *n*=6, resistant (PD-1R): *n*=9). (**c**) Significant correlation was detected among TIM-3 positivity and the amount of bound therapeutic PD-1 antibody in anti-PD-1 resistant (PD-1R) EGFR TL and Kras mice (both EGFR and Kras mice were combined: *n*=18). Correlation was evaluated using Pearson's correlation coefficient.

To further investigate TIM-3 expression in T cells, we systemically analysed mice at the time of resistance to PD-1 blockade. TIM-3 upregulation was only detected specifically in T cells from tumour-bearing lungs but not mediastinal lymph node, peripheral blood (Fig. 2a) or spleen (data not shown) and was predominantly found on anti-PD-1 antibody bound CD4 and CD8 T cells (Fig. 2a). We also evaluated the kinetics of TIM-3 upregulation during PD-1 blocking treatment. We previously showed that significant T-cell activation and clinical response could be observed in mouse models following 1 week of anti-PD-1 therapy^17^. At this time point, there was no significant difference in TIM-3 expression between treated and untreated tumours in both EGFR and Kras mice; however, a significant increase in interferon-gamma-positive CD8 T cells was observed (Fig. 2b, Supplementary Fig. 1a,f) suggesting that TIM-3 elevation was not simply correlated with T-cell activation. In contrast, significant TIM-3 upregulation was detected at the time of disease progression (PD-1R) in both models (after 2 weeks in Kras and after 4 weeks in EGFR TL) and there were significant correlations between TIM-3 positivity and the duration of PD-1 blocking treatment (Fig. 2b) and the percentage of anti-PD-1 antibody positive T cells (Fig. 2c). Together with the finding that anti-PD-1 antibody binding was increased with longer duration of PD-1 blocking treatment (Supplementary Fig. 1e), these results suggested that therapeutic PD-1 blockade could facilitate persistence of TILs with enrichment for therapeutic antibody bound PD-1- and TIM-3-positive T cells. In contrast, we did not see TIM-3 upregulation with CTLA-4 blockade.

We also investigated the expression of Galectin-9, one of the ligands for TIM-3 receptor that is expressed on a variety of cell types and has a role in negatively regulating Th1-type immune responses^20^,^21^. We confirmed a significant elevation in the expression of lectin, galactoside-binding, soluble, 9 (*Lgals9)*, which encodes Galectin 9, in sorted CD45^−^EpCAM^+^ tumour samples from the anti-PD-1-resistant Kras tumours as compared with untreated Kras tumours at both RNA and protein level (Supplementary Fig. 2a,b). CEACAM1 and phosphatidylserine have also been proposed as ligands for TIM-3 (refs 22, 23). We did observe that the majority of TIM-3-positive CD8 T cells co-expressed CEACAM1 (Supplementary Fig. 3a) suggesting at the time of resistance to PD-1 blockade that TIM-3 might function together with CEACAM1 to suppress T cells. CEACAM1 was also expressed in some TIM-3-negative T cells and tumour cells. The level of CEACAM1 expression in tumour cells and positivity of CEACAM1 in CD4 and CD8 T cells did not show a significant difference between untreated and PD-1 treatment-resistant tumours (Supplementary Figs 2b,3b).

### TIM-3 antibody addition overcomes resistance to PD-1 blockade

**Figure 3:**
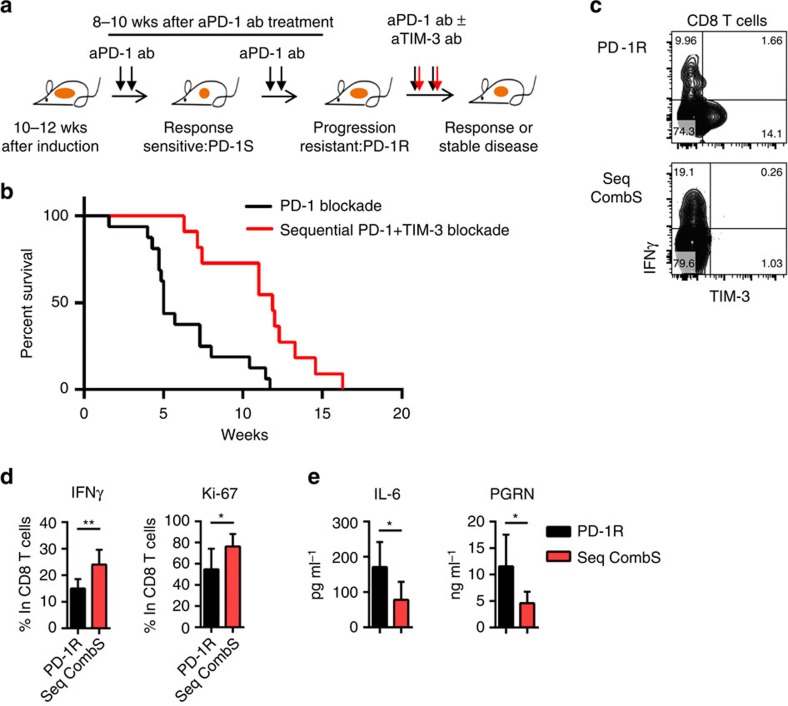
Sequential anti-TIM-3 blocking displays clinical efficacy in anti-PD-1 adaptive resistant tumours. (**a**,**b**) Survival after PD-1 blockade alone (anti-PD-1 resistant) or PD-1 and sequential TIM-3 blockade combination treatment (PD-1 alone: *n*=16 and sequential combination treatment: *n*=11) (*P*=0.0008) after documented tumor burden. Treatment started at week 0. Median survival PD1 5 weeks vs PD-1+TIM-3 sequential treatment 11.9 weeks. (**c**) Representative flow cytometry data of IFN-gamma expression in CD8 T cells from anti-PD-1 resistant (PD-1R) and sequential anti-PD-1 plus anti-TIM-3 combination (Seq CombS): 2 weeks′ anti-PD-1 and anti-TIM-3 combination treatment after development of resistance to PD-1 single treatment. Fluorescent conjugated anti-TIM-3 antibody is the same clone (RMT3-23) as the therapeutic antibody. (**d**) IFN-gamma and Ki-67 positive CD8 T cell counts from anti-PD-1 resistant (PD-1R) (*n*=6) and sequential anti-PD-1 plus anti-TIM-3 combination (Seq CombS) (*n*=6) (*P<0.05, **P<0.01). (**e**) IL-6 and PGRN production in BALFs from PD-1R (*n*=6) and comb (Seq CombS: *n*=6) (*P<0.05). Data are shown as mean±s.d., *P* values are calculated using student's *t* test for all data except for the survival data.

As we observed PD-1 blocking antibody on TILs as well as TIM-3 upregulation at the time of therapeutic resistance to anti-PD-1 therapy, we treated mice with an anti-TIM-3 antibody at the time of PD-1 treatment failure to investigate whether TIM-3 blockade could provide additional clinical benefit in tumours, which had developed resistance to anti-PD-1 treatment. Because the clinical response to anti-PD-1 antibody was initially more robust in the EGFR TL model^17^ (Supplementary Fig. 4a) as compared with the Kras model that only showed disease stability with PD-1 treatment, we utilized the EGFR model for therapeutic studies with a TIM-3-blocking antibody. Mice were treated with a PD-1 blocking antibody until tumours progressed and, then treatment with a TIM-3-blocking antibody was initiated when mice appeared both clinically unwell and demonstrated progressive disease by MRI imaging (Fig. 3a, Supplementary Fig. 4b). Antitumour efficacy (Supplementary Fig. 4b) and a significant survival advantage (Fig. 3b) were observed in the cohort of mice that were treated with the TIM-3-blocking antibody with median survival for anti-PD-1 antibody alone 5 weeks versus PD-1+TIM-3 sequential treatment 11.9 weeks (Fig. 3b; *P*=0.0008, log-rank test). The impact of the TIM-3-blocking antibody on T-cell function was investigated by analysing mice at 2 weeks after adding TIM-3 blockade to anti-PD-1 antibody therapy (sequential combination treatment sensitive:Seq combS; Supplementary Fig. 5a). Binding of both anti-PD-1- and anti-TIM-3-blocking antibodies on CD8 T cells was confirmed. Addition of TIM-3-blocking antibody but not an isotype control antibody (Rat IgG2a) enhanced interferon-gamma production and cell proliferation as compared with TIM-3^+^ CD8 T cells from PD-1-resistant mice (Fig. 3c,d). Importantly, we also detected higher levels of LAG-3 and CTLA-4 on the CD8 T cells that were bound by the anti-PD-1 and anti-TIM-3 antibodies at the time of regrowth of lung tumour after combination treatment (Seq combR) as compared with the time of inhibiting lung tumour growth (Seq combS) (Supplementary Fig. 5b,c). This result suggests that additional immune checkpoints may be upregulated in the context of combinatorial therapy with anti-PD-1 and anti-TIM-3 antibodies, which also might limit therapeutic activity.

As we reported previously, checkpoint blockade also affected immune suppressive cytokine production in the tumour microenvironment^17^. We found that IL-6 and progranulin (PGRN) were significantly reduced with combined anti-PD-1 and anti-TIM-3 treatment following anti-PD-1 antibody failure as compared with the levels at the time of anti-PD-1 resistance (Fig. 3e). This result suggests that TIM-3 blockade may not only enhance T-cell function following anti-PD-1 antibody failure, but also decrease the levels of tumour-promoting cytokines, similar to our previous observation in naive mice treated with anti-PD-1 alone^17^. Unlike other models where concurrent blockade of PD-1 and TIM-3 delays tumour progression as initial therapy in the context of high expression levels of these checkpoints^24^, we observed no additional benefit of combination therapy as initial therapy as compared with anti-PD-1 alone (Supplementary Fig. 6a,b). Although the precise basis for this difference remains to be clarified, low levels of TIM-3 expression at the time of treatment initiation and the rapid development of neutralizing anti-rat antibodies might be involved.

### High TIM-3 expression in TILs is observed in patients

To assess whether these findings in mouse models might correspond to patterns of resistance to anti-PD-1 therapy in lung cancer patients, we analysed samples from two lung cancer patients who were treated with anti-PD-1 antibodies and five samples from lung cancer patients who were not treated with immune modulating agents.

**Figure 4:**
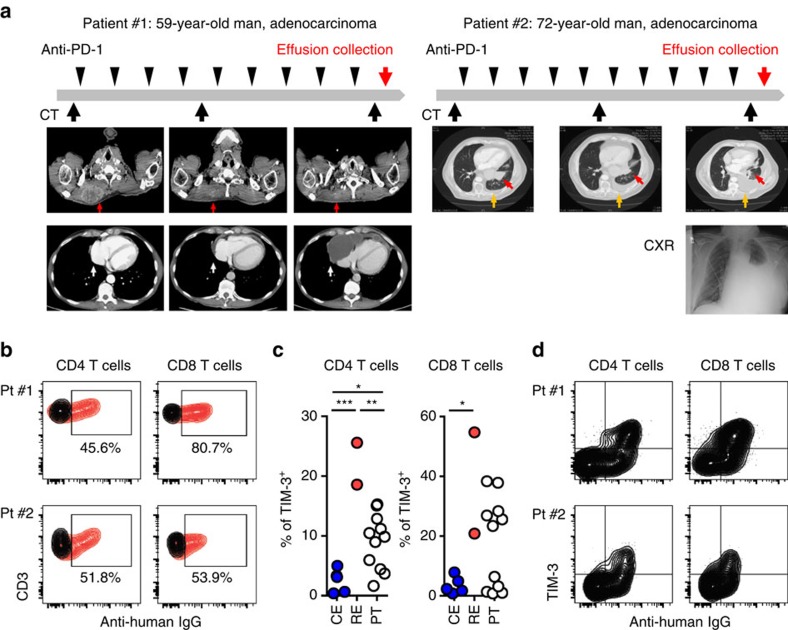
Upregulation of TIM-3 in two resistant patient cases after anti-PD-1 treatment. (**a**) Clinical course of patient #1 and patient #2, who were treated with PD-1 blocking antibodies. Both initially responded to treatment but subsequently developed treatment resistance with effusions. Arrow indicates soft tissue metastasis (red arrow) and pericardial effusion (white arrow) in patient #1 and left lower lobe tumour (red arrow) and pleural effusion (orange arrow) in patient #2. (**b**) Detection of therapeutic antibody (human IgG) binding in CD4 and CD8 T cells. Human IgG and isotype control are shown in red and black, respectively. (**c**) Percentage of TIM-3 positive CD4 and CD8 T cells in effusions from two anti-PD-1 resistant patients (resistant effusions: RE), NSCLC patients without PD-1 blocking treatment (control effusions: CE, *n*=5) and surgically resected primary tumours: PT (*n*=11). Mean % of TIM-3 in T cells from RE versus CE versus PT (CD4 T cells 22.10 versus 2.52 versus 9.06 and CD8 T cells 37.85 versus 3.19 versus 17.58. In CD4 T cells, RE versus CE ***P=0.0001, RE versus PT **P=0.0023 and CE versus PT *P=0.0247. In CD8 T cells, RE versus CE *P=0.0256. (**d**) TIM-3 expression and therapeutic antibody binding (human IgG) in CD4 and CD8 T cells. Data are shown as mean±s.d., *P* values are calculated using one-way analysis of variance with Tukey's multiple comparison test.

Patient #1 was a 59-year-old male with an extensive smoking history who harboured a *KRAS* G12D mutated stage IV lung adenocarcinoma with diffuse metastases. Following progression on carboplatin, paclitaxel and bevacizumab therapy, he was enrolled in a clinical trial of anti-PD-1 therapy at which time his tumour was determined to be PD-L1 positive by IHC (>1% positivity). The patient achieved a partial response to this treatment as defined by RECIST 1.1 criteria but developed progressive disease 4 months later with a new pericardial effusion (Fig. 4a). Patient #2 was a 72-year-old male former smoker initially diagnosed with stage IV lung adenocarcinoma with brain metastases. His tumour was wild type for EGFR, KRAS and ALK, but did display MET positivity by IHC. He was treated with carboplatin and paclitaxel, pemetrexed and erlotinib plus an anti-MET antibody before enrolling on a study of anti-PD1 therapy. His tumour displayed >50% PD-L1 positivity at the time of trial registration. He achieved a partial response of 5 months duration before developing disease progression with an enlarging parenchymal lung mass and a malignant pleural effusion (Fig. 4a). We analysed the immune cells in the effusion samples collected from these two patients and compared the immune profile with effusions and surgically resected tumour samples from different NSCLC patients who had not been treated with anti-PD-1 antibody treatment.

At the time of disease progression, CD4 and CD8 T cells in effusions from both anti-PD-1 treated patients showed evidence of therapeutic antibody binding (more than 45% of CD4 and CD8 T cells showed human IgG binding; Fig. 4b), indicating that PD-1 was still expressed by these T cells at the time of treatment failure. On detailed analysis of other immune checkpoints (TIM-3, LAG-3 and CTLA-4) and the regulatory T cell marker FOXP3 on the T cells, we detected upregulation of TIM-3 but no significant changes in the other markers examined as compared with effusions from other lung cancer patients who had not been treated with PD-1-blocking antibodies (Fig. 4c, Supplementary Fig. 7a). These results show a similar trend to what was observed in mouse models (Fig. 1d). There was also an increase of CTLA-4 expression in CD8 T cells in the two resistant cases (Supplementary Fig. 7a), but the magnitude of this change was less than that observed for TIM-3, also consistent with the mouse data (Fig. 1d). Although tumour-infiltrating T cells from surgically resected primary NSCLCs (PT) showed variation in TIM-3 expression, the level of TIM-3 on CD4 and CD8 T cells in the effusion specimens from the two patients who progressed on anti-PD-1 therapy (resistant effusions: RE) was higher (mean of TIM-3 positivity was 22.10% and 37.85% in CD4 and CD8 T cells, respectively) than those from other NSCLC patients (mean of TIM-3 positivity was control effusions (CE):2.52% and PT:9.06% in CD4 T cells and 3.54% and PT:17.58% in CD8 T cells) (Fig. 4c). Further analysis of the TIM-3 expressing T-cell population in the patients who progressed on anti-PD-1 therapy showed that the majority of TIM-3 expressing T cells bound the therapeutic antibody (human IgG^+^; Fig. 4d). Since previous work has indicated that TIM-3^+^FOXP3^+^ regulatory T cells possess strong immunosuppressive functions in mice^25^, we also evaluated the PD-1 antibody binding and TIM-3 expression specifically in FOXP3^+^CD4 T cells; TIM-3 expression in Tregs was similarly linked to anti-PD-1 antibody binding (Supplementary Fig. 7b). In accordance with the finding in mouse models, TIM-3 levels correlated with PD-1 expression in tumour-infiltrating cytotoxic CD8 T cells from surgically resected NSCLCs (Supplementary Fig. 7c). Together, with the findings in preclinical mouse models, our data suggest that prolonged exposure to PD-1 blocking antibody drives increased TIM-3 expression in NSCLC patients, which might impact overall treatment efficacy.

We further investigated T cells and myeloid cell populations in effusion samples. The CD4/CD8 ratio was lower in T cells at resistance in both patients (Supplementary Fig. 7d), as it was the case for the mouse EGFR TL and Kras lung cancer models (Fig. 1a) In the anti-PD-1-resistant samples, we noted increased effector memory CD8 T cells (CCR7^−^CD45RA^−^) as compared with untreated samples (Supplementary Fig. 7d). Together with other work^6^, these results suggest that PD-1-blocking treatment prolongs T-cell survival; however, PD-1 blockade also enriches for tumour-reactive memory CD8 T cells expressing TIM-3. Two major myeloid cell populations (CD66b^+^ and CD33^+^CD66b^−^) did not show a significant difference between untreated and anti-PD-1-resistant samples (Supplementary Fig. 7e). We also evaluated PD-L1 expression levels in human monocytes (CD33^+^CD66b^−^CD14^+^) and found a nonsignificant trend towards increased PD-L1 expression in the anti-PD-1-resistant samples (Supplementary Fig. 7f). To evaluate cytokine production in tumour microenvironment, we also analysed supernatants from the effusion samples. We detected comparable levels of IL-6 and PGRN in both resistant samples and untreated effusion samples, similar to our observation in the mouse models. (Supplementary Fig. 7g). Interestingly, Galectin-9 (ref. 20), which was significantly increased in mouse cancer models (Supplementary Fig. 3) was also significantly increased in both of the PD-1-resistant patient cases as compared with untreated samples (Supplementary Fig. 7g). Together, these results suggest that TIM-3 blockade could be a reasonable therapeutic option in the setting of resistance to anti-PD-1 therapy because both TIM-3 on T cells and ligand in the tumour microenvironment showed significant increases in PD-1-resistant clinical cases.

## Discussion

Previously we demonstrated that oncogenic signalling downstream of the EGFR kinase in a lung cancer mouse model and in human cancer cell lines contributed to immune evasion through the activation of the PD-1:PD-L1 immune checkpoint. We functionally validated the therapeutic efficacy of PD-1 blockade in this mouse model and showed that suppression of EGFR signalling decreased PD-L1 expression on tumour cells^17^. Here, we have extended these studies to investigate potential mechanisms of adaptive resistance to anti-PD-1 therapy using two fully immunocompetent genetically engineered mouse models of lung adenocarcinomas corresponding to the two most common oncogene drivers in human lung adenocarcinoma, *KRAS* and *EGFR*. This study was motivated by the impressive efficacy of anti-PD-1:PD-L1 therapy in lung cancer patients, which has been recently approved by FDA.

Here, we showed that the therapeutic PD-1-blocking antibody was still bound to T cells at the time of disease progression in both EGFR and Kras mutated mouse lung tumours as well as in human specimens. These data indicate that anti-PD-1 antibodies are still functional at the time of resistance and maintain the ability to prevent inhibitory signals through PD-1 in the tumour microenvironment and suggest that mechanisms other than PD-1:PD-L1 interaction are likely important in adaptive resistance. This argues against failure of ongoing antibody binding to its target as a mechanism of treatment failure. Further, we did not observe a significant difference in the number of tumour-associated macrophages or tumour-associated neutrophils when comparing pre-treatment and resistant specimens, arguing against quantitative changes in myeloid cell composition as likely resistance mechanisms. We demonstrated that TIM-3 was upregulated in CD4 and CD8 T cells from anti-PD-1-resistant EGFR and Kras mutated tumours as compared with untreated tumours. Importantly, (1) elevation of TIM-3 expression in T cells was predominantly found on the therapeutic PD-1 antibody-bound subset, (2) upregulation of TIM-3 was only detected in tumour-bearing lungs but not in sentinel lymph nodes and peripheral blood, (3) TIM-3 positivity was significantly correlated with duration of PD-1 blockade and (4) TIM-3 was not upregulated acutely at time points when we confirmed clinical efficacy^17^. Other studies in the basic Immunology and Infectious Disease literature have shown that TIM-3 is co-expressed with PD-1 in exhausted T cells in the context of anti-microbial responses^26^,^27^,^28^,^29^, which support our finding of TIM-3 expression on therapeutic anti-PD-1 antibody-bound T cells in the setting of treatment failure. In mice lacking PD-1 systemically, T cells express higher levels of TIM-3 in the context of AML^30^ supporting the notion that compensatory pathways are upregulated in the absence of PD-1. Together, with these studies, our results suggest that prolonged exposure to PD-1-blocking antibody enriches for TIM-3 expression in therapeutic antibody-bound T cells specifically in the tumour microenvironment and not systemically; and additional inhibitory signals through TIM-3 can limit tumour-reactive T cell function in anti-PD-1-resistant tumours. Although previous studies have reported the synergistic efficacy of combinational anti-PD-1 plus anti-TIM-3 treatment in xenograft and carcinogen-induced tumour models^31^, we provide here the first evidence showing the efficacy of TIM-3 blockade following anti-PD-1 therapy failure in genetically engineered mouse model in the context of adaptive resistance, which reproduces a more clinically relevant situation as demonstrated by our two case vignettes^1^,^32^.

Interestingly, surgically resected human tumour samples show that TIM-3 expression varies even in PD-1-high T cells in treatment naive patients. This may suggest that expression of immune checkpoints beyond PD-1:PD-L1 is important in determining response to anti-PD-1 therapy. Given that only a minority of lung cancer patients respond to anti-PD-1 (ref. 12), it would be of interest to correlate expression of other checkpoints at the time of treatment initiation with response to PD-1 blockade. Although we report no benefit in the EGFR TL mouse model of concurrent PD-1 plus TIM-3 blockade, we would caution against extrapolation of this result to human lung cancer given that this model expresses scant TIM-3 before PD-1 treatment and develops neutralizing antibodies to therapy which should not be the case in humans treated with humanized therapeutic antibodies. Genetically engineered mouse models are genetically heterogeneous and therefore unlikely to manifest all of the potential mechanisms associated with resistance to PD-1 blockade and specifically are not suitable for the study of immune editing of specific tumour neoantigens or loss of antigen presentation as potential resistance mechanisms to anti-PD-1 therapy^33^, processes which will likely require evaluation in large cohorts of primary patient specimens.

Our data suggest that targeting specific immune checkpoints engaged by lung tumours, as identified by flow cytometry and/or gene expression analysis, may represent a rational approach to the selection of specific immunotherapy regimens. Given the current clinical interest in building upon the success of anti-PD-1 therapy in lung cancer, we feel that direct measurement of multiple checkpoints could allow for the development of targeted immunotherapy approaches in selected patients based on direct analysis of immune checkpoint biomarkers. We also feel that the specific immune checkpoints engaged will likely be impacted by cancer therapies, as we observe with TIM-3 and LAG-3 upregulation following PD-1 blockade, and that serial measurements will be necessary to best understand the status of the tumour immune microenvironment and to aid the selection of appropriate immunotherapies.

Our data also suggest, as is well known in the kinase inhibitor field, that cancers possess the ability to evolve in the face of immunotherapy and to escape therapy by engaging bypass pathways. In the cases studied in this report, we observed upregulation of not only TIM-3 on T cells but Galectin-9 on tumour cells in the case of Kras mutant tumours, indicating that resistance to PD-1 therapy can be driven by coordinated interactions among the tumour and neighbouring immune cells. When larger cohorts of patient samples are analysed, they will very likely display heterogeneous patterns of suppressive mechanisms at the time of resistance, possibly including changes in the T-cell suppressive cytokines or in myeloid-derived suppressor cells, compensatory upregulation of additional immune checkpoints as seen here or immune editing in tumour cell populations. Understanding the dynamics and diversity of these mechanisms will identify therapeutic strategies that can prolong the efficacy of immunotherapy and thereby improve patient outcomes.

## Methods

### Mouse treatment studies

EGFR transgenic mice carrying tetracycline-inducible human EGFR cDNA were previously generated^34^. L858R T790M mutation was generated by site-directed mutagenesis of the pCIBA-*hEGFR* plasmid. The fragment containing the whole *hEGFR* ORF with the Kozak site was then subcloned into pTRE2-hyg (Clon- tech, Mountain View, CA). The constructs were then digested to release the entire allele containing *Tet-op-EGFR TL*-b*-globin* polyA. Transgenic mice were then generated by injection of the construct into FVB/N blastocysts. EGFR mutant mice were crossed with CC10-RTTA mice expressing reverse tetracycline activator from the lung Clara cell CC10 promoter, and maintained in mixed background. Double-positive (EGFR and CC10 RTTA) progeny were fed with a doxycycline diet starting at 5–6 weeks of age for the induction of tumours and maintained on doxycycline throughout the study. Kras G12D mice were given adenovirus expressing Cre recombinase (5 × 10^6^ titre) intranasally at 5 weeks of age for induction of recombination and tumour formation^35^. All the mice were maintained on a mixed (C57Bl/6, FVB and S129) background. The mice were euthanized when they reached tumour burden euthanasia criteria determined by health condition as evaluated by veterinary technicians upon twice daily health checks. TIM-3 antibody was added to the treatment regimen when mice displayed clinical signs of progressive disease, which was confirmed by MRI. All breedings and *in vivo* experiments were performed with the approval of the DFCI Animal Care and Use Committee. MRI imaging was performed using the 7 Tesla (BioSpec: Bruker BioSpin) MRI. Tumour volume quantifications were performed using the 3D-Slicer software. PD-1-blocking antibody (clone 29F.1A12), TIM-3-blocking antibody (clone RMT3–23: Bio X cell) and their isotype controls (clone 2A3: Bio X cell) were injected intraperitoneally into the mice for therapeutic treatment (three times a week, 200 μg for PD1 and 100 μg for TIM-3 per dose).

### Patient sample collection

Anonymized patient samples were obtained under IRB approved protocols 02–180 and 11–104 and BIDMC 2001-P-001089 from subjects providing informed consent for tissue collection. Biopsies and effusions were obtained during routine clinical procedures. All human subjects research was performed in accordance with the above protocols approved by the Institutional Review Boards at the Dana-Farber Cancer Institute and Beth Israel Deaconess Medical Center.

### Immune analysis for patient and mouse samples

Murine tumour and immune cell characterization was performed as previously described^17^ and detailed in Supplementary Methods. The processing for freshly resected patient lung tumour samples was performed similarly. For freshly collected effusion samples, the cells were treated with RBC lysis after spin and directly used for staining after cell screening (70 μm). Isolated cells were stained with LIVE/DEAD fixable dead cell stain kit (Invitrogen) before surface marker staining. The antibodies used for immune analysis are listed in the Supplementary Appendix. For counting absolute numbers of immune cell populations, AccuCheck Counting Beads (Molecular probes) were used according to the manufacturer's protocol. For detecting anti PD-1 antibody binding, Rabbit anti human IgG/Rabbit isotype control IgG (SouthernBiotech) and secondary Goat anti Rabbit IgG (SouthernBiotech) for human and anti Rat IgG2a (r2a-21B2: eBioscience) for mice were used without prior Fc blocking (Miltenyi Biotech and BD Biosciences), which was used for all the other staining. All antibodies were used at 1:50 dilution. For intracellular cytokine staining, total tumour-bearing lung cells were fractionated over cell separation media, OptiPrep (Sigma) and buffered saline with Tricine (Sigma) as per the manufacturer's instructions (Axis-Shield, Application Sheet C43). Isolated mononuclear cells were stimulated with 50 ng ml^−1^ PMA (Sigma) and 500 ng ml^−1^ Ionomycin (Sigma) for 4 h in the presence of Golgi plug (BD Biosciences). Fixation/permeabilization buffers (eBioscience) or BD Cytofix/Cytoperm buffers (BD Biosciences) were used for both mice and human samples for intracellular staining. Acquisition of eight colour samples was performed on a BD Canto II cytometer equipped with Diva software and analysed using Flowjo. Antibody clone numbers for mouse are provided in Supplementary Table 1 and for human in Supplementary Table 2.

### Tumour-infiltrating T-cell sorting and RNA sequencing

Sorting of tumour-infiltrating T cells (CD45^+^TCRb^+^CD11b^−^CD11c^−^CD19^−^DX5^−^TER119^−^Ly6G^−^) and tumour cells (enriched epithelial cell population: CD45^−^EpCAM^+^ was utilized as tumour cells) was performed on a BD FACSAria II cell sorter. The gating method for sorting is shown in Supplementary Fig. 8. RNA was prepared from sorted lymphocyte populations using the Arcturus PicoPure kit (Life Technologies) and RNA quantified using Ribo-Green (Life Technologies) per the manufacturer's protocol. 10 ng of total RNA was used for library preparation using the Nugen Ovation system (Nugen) as per the manufacturer's instructions. Libraries were quantified and analysed using a high-sensitivity DNA chip assay (Agilent) and by quantitative PCR. Pooled libraries were sequenced on an Illumina HiSeq instrument to a minimum read depth of 30 million reads. RNA-seq reads were aligned to the mm9 Ensembl transcript annotation (release 65) using the PRADA pipeline (10.1093/bioinformatics/btu169), and FPKM expression values were determined using Cufflinks^36^ with mm9 RefSeq gene annotations and subsequently log2-transformed. Expression values of T-cell samples were normalized to the expression value of Cd3e in each respective sample and tumour samples were similarly normalized to Epcam. These normalized expression values of resistant and untreated tumours were used to calculate fold change and input into the limma package (PMID: 25605792) to calculate *P* values based on a moderated *t*-statistic. Differentially expressed genes were defined as those with absolute fold change over 1.25 and *P* value under 0.05. For heatmaps, the log2-transformed FPKM values were row-scaled and coloured on a blue-red scale ranging from −2 to 2. Data can be accessed with the BioProject number PRJNA305565 at the NCBI BioProject database.

### Cytokine and chemokine measurements

BALF collection was performed by injecting 1 ml of PBS into the trachea to inflate the lungs, which were then aspirated. Collected BALFs and supernatants of effusions were kept at −80° before performing the ELISA. Cytokine and chemokines were measured with ELISA kits according to the manufacturer's protocol; mouse and human IL-6 (BD Biosciences), GRN (R&D Systems) and human Galectin-9 (R&D Systems).

### Statistical analysis

All numerical data are shown as mean±s.d. Data were analysed using two-tailed unpaired Student's *t*-test for comparisons of two groups and one-way analysis of variance with Tukey multiple comparison test for three groups. Correlation was evaluated using Pearson's correlation coefficient. *P* values for the survival curves have been calculated using a log-rank test.

## Additional information

**Accession codes:** The RNA-seq data have been deposited in the NCBI BioProject database under accession code PRJNA305565.

**How to cite this article:** Koyama, S. *et al*. Adaptive resistance to therapeutic PD-1 blockade is associated with upregulation of alternative immune checkpoints. *Nat. Commun.* 7:10501 doi: 10.1038/ncomms10501 (2016).

## Supplementary Material

Supplementary Information Supplementary Figures 1-8, Supplementary Tables 1-2 and Supplementary Methods.
