# Supplementary material for: Adaptive resistance to therapeutic PD-1 blockade is associated with upregulation of alternative immune checkpoints
Source: Nat Commun. 2016 Feb 17;7:10501. doi: 10.1038/ncomms10501 (PMC4757784; doi:10.1038/ncomms10501)
Supplement: Supplementary Information — Supplementary Figures 1-8, Supplementary Tables 1-2 and Supplementary Methods. [file ncomms10501-s1.pdf]

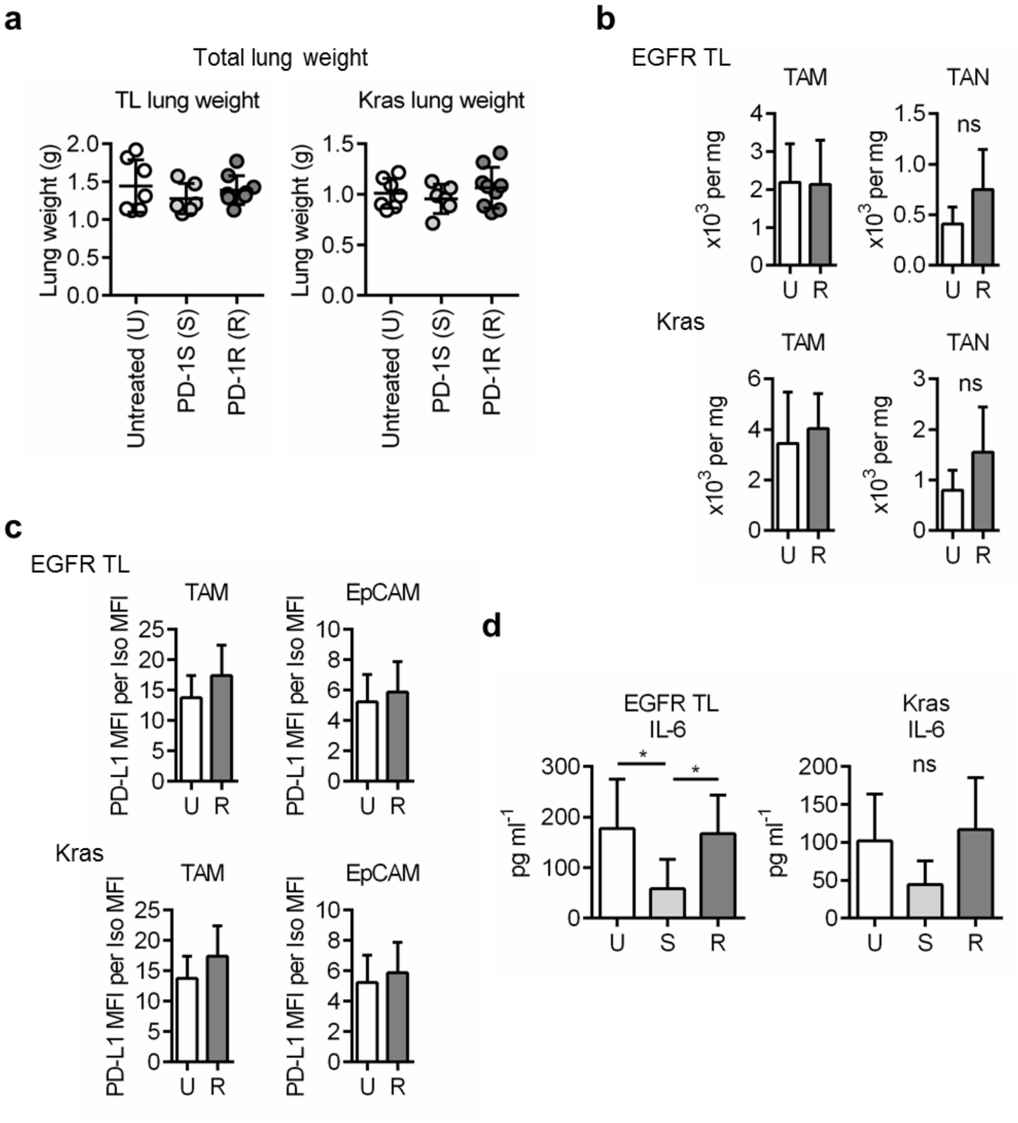
a
Total lung weight
TL lung weight
Kras lung weight
Lung weight (g)
2.0
1.5
1.0
0.5
0.0
Untreated (U)
PD-1S (S)
PD-1R (R)
b
EGFR TL
TAM
TAN
ns
x10^3 per mg
U R
Kras
c
EGFR TL
TAM
EpCAM
PD-L1 MFI per Iso MFI
Kras
d
EGFR TL
IL-6
Kras
IL-6
ns
pg ml^-1
*
U S R

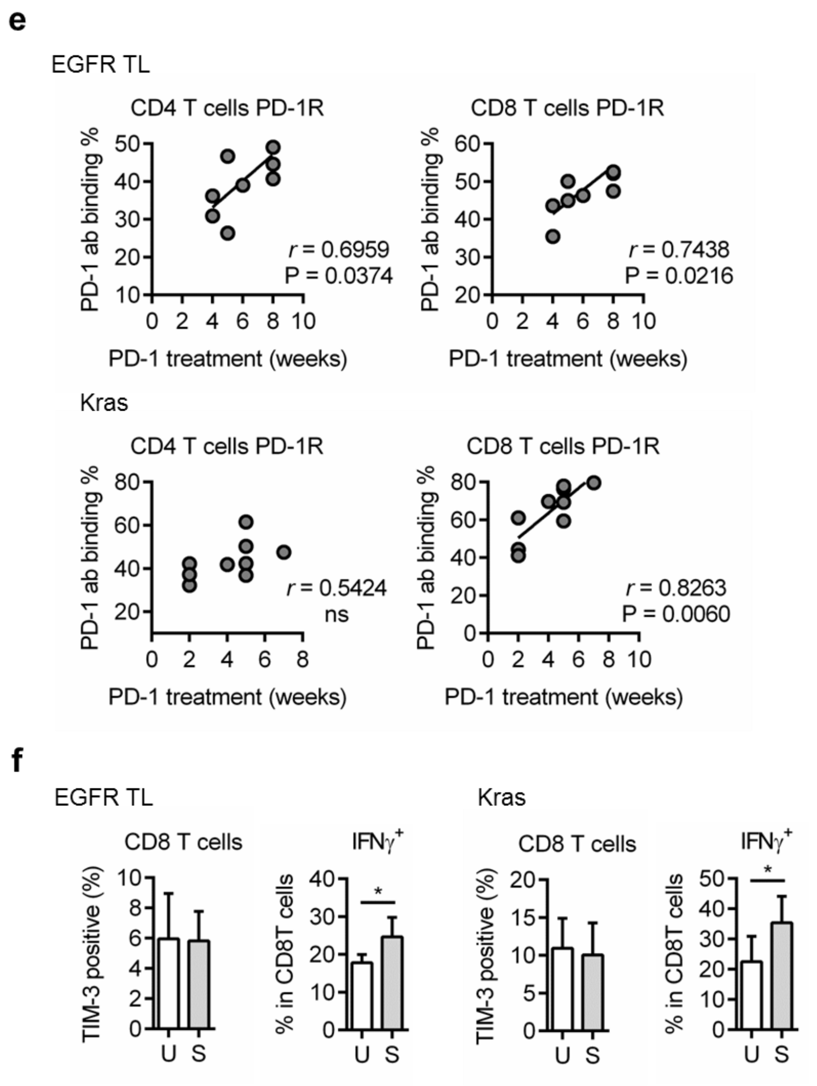

Supplementary Figure 1. Immune profiles of untreated and PD-1 blockade resistant EGFR and Kras mouse lung tumors

(a) Total lung weight of untreated (U) EGFR TL mice (n=7), Kras mice (n=7), PD-1 blockade sensitive (S) EGFR TL mice (n=6), Kras mice (n=6) and resistant (R) EGFR TL mice (n=9), Kras mice (n=9). (b) Numbers of myeloid cells in EGFR TL (U:n=7, R:n=9) and Kras lung tumors (U:n=7, R:n=9). TAM: tumor associated macrophage, TAN: tumor associated neutrophil. (c) Mean of fold expression of PD-L1 MFI (mean fluorescent intensity) in tumor-associated macrophages (TAM) and tumor cells ($CD45^{-}EpCAM^{+}$) from EGFR TL (U:n=7, R:n=9) and Kras (U:n=7, R:n=9) lung tumors. (d) IL-6 production in BALFs from untreated (U) EGFR TL mice (n=7), Kras mice (n=7), PD-1S (S) EGFR TL mice (n=6), Kras mice (n=6) and PD-1R (R) EGFR TL mice (n=9), Kras mice (n=9). (e) Plots showing the relationship between therapeutic PD-1 antibody binding in CD4 and CD8 T cells and the duration of

treatment in PD-1 blockade resistant EGFR TL lung tumors (n=9) and Kras lung tumors (n=9). (f) TIM-3 expression and IFNγ production in CD8 T cells from EGFR TL mice (untreated: n=7, sensitive (1wk after treatment): n=6) and Kras (untreated: n=7 and sensitive: n=6). Data are shown as mean ± standard deviation (b,c,d,f). Statistical analysis was performed using Student's *t* test (b,c,f), one-way ANOVA with Tukey's multiple comparison test (d) and Pearson's correlation coefficient (e).

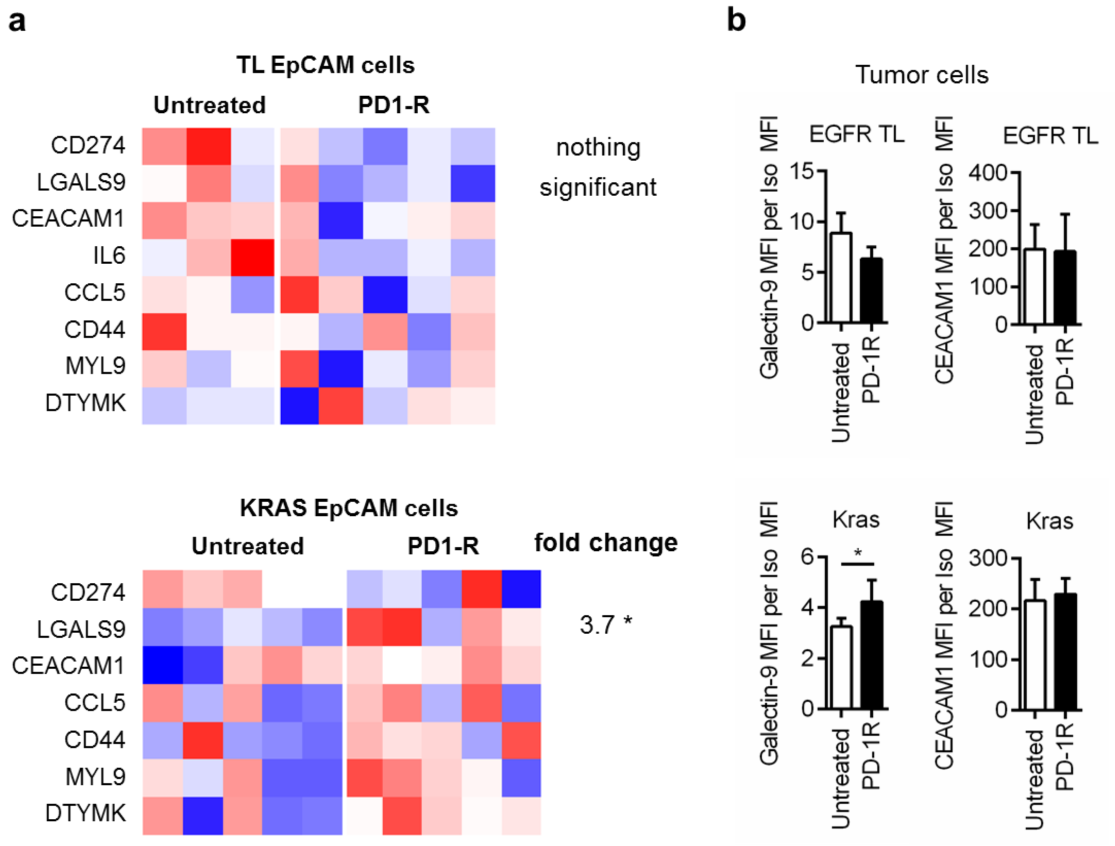

Supplementary Figure 2. The expression of Galectin-9 and CEACAM1 in untreated vs PD-1 resistant tumors

(a) RNAseq analysis of $CD45^{-}EpCAM^{+}$ tumor cells from PD-1 naïve or PD-1 resistant tumors showing expression of selected immune related markers (*p=0.02, statistical analysis was performed using Limma) (b) Mean of fold expression of Galectin-9 and CEACAM1 in EGFR TL (untreated:n=3 and PD-1R:n=3) and Kras tumor cells (untreated:n=3 and PD-1R:n=3) shown as MFI (mean fluorescent intensity). Data are shown as mean ± standard deviation. *P<0.05, Student's t test.

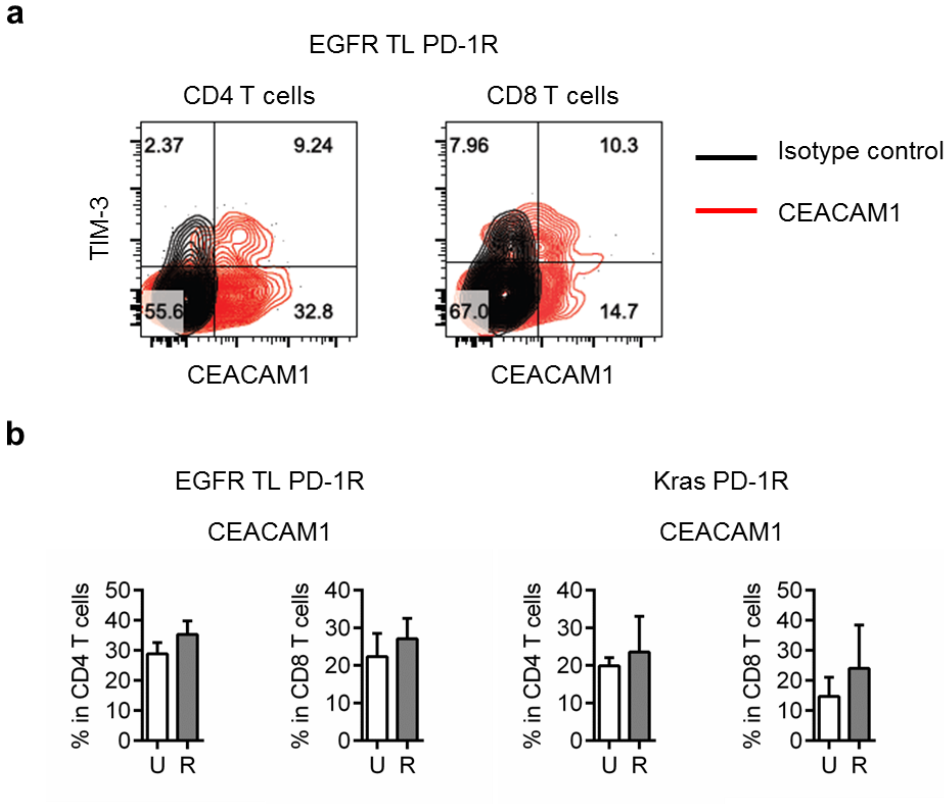

Supplementary Figure 3. Co-expression of TIM-3 and CEACAM1 in T cells from PD-1 resistant tumors

(a) Representative flow cytometry data of anti-PD-1 resistant EGFR TL tumors for CEACAM1 and TIM-3. Experiments were repeated three times. (b) Levels of CEACAM1 in CD4 and CD8 T cells as determined by flow showed no significant difference between untreated (EGFR TL:n=4 and Kras:n=5) and PD-1 resistance (EGFR TL:n=5 and Kras;n=6). Data are shown as mean ± standard deviation.

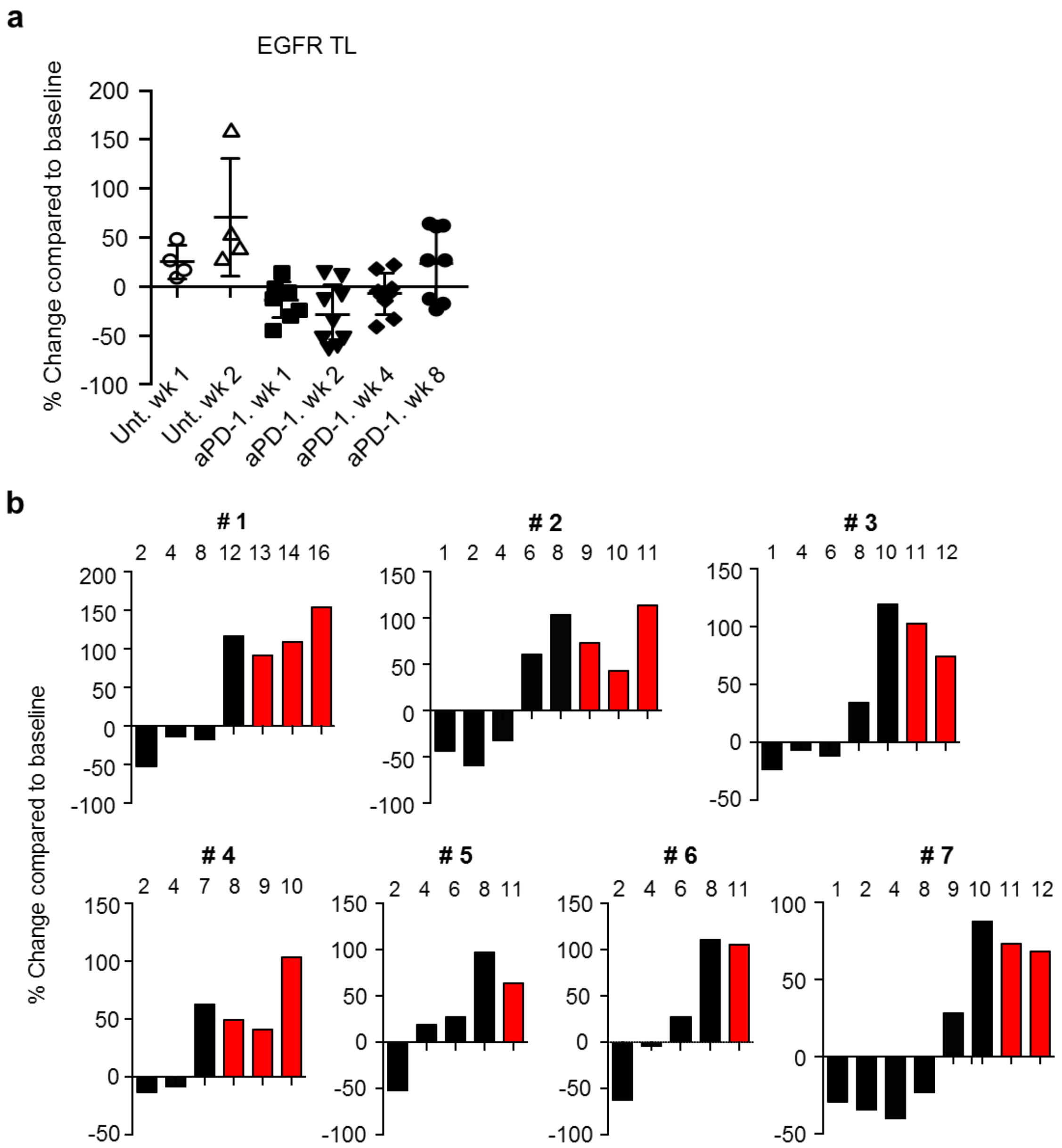

Supplementary Figure 4. Tumor volume measurements from mice treated with anti-PD-1 alone and anti-PD-1 plus sequential TIM-3 blockade

(a) MRI tumor volume measurements of all mice treated in the PD-1 and Tim3 sequential treatment study. Each data point represents a different mouse at that time point.

(b) Waterfall plots of MRI tumor volume measurements for individual EGFR TL mice treated in the anti-PD-1 and anti-TIM-3 sequential treatment study. Mouse ID numbers are indicated on top of each graph and each data point represents MRI measurement at the indicated time points (in weeks) indicated on top of the columns. Black bars show tumor volumes on anti-PD-1 treatment, red bars show tumor volumes after anti-TIM-3 is added to the anti-PD-1 treatment.

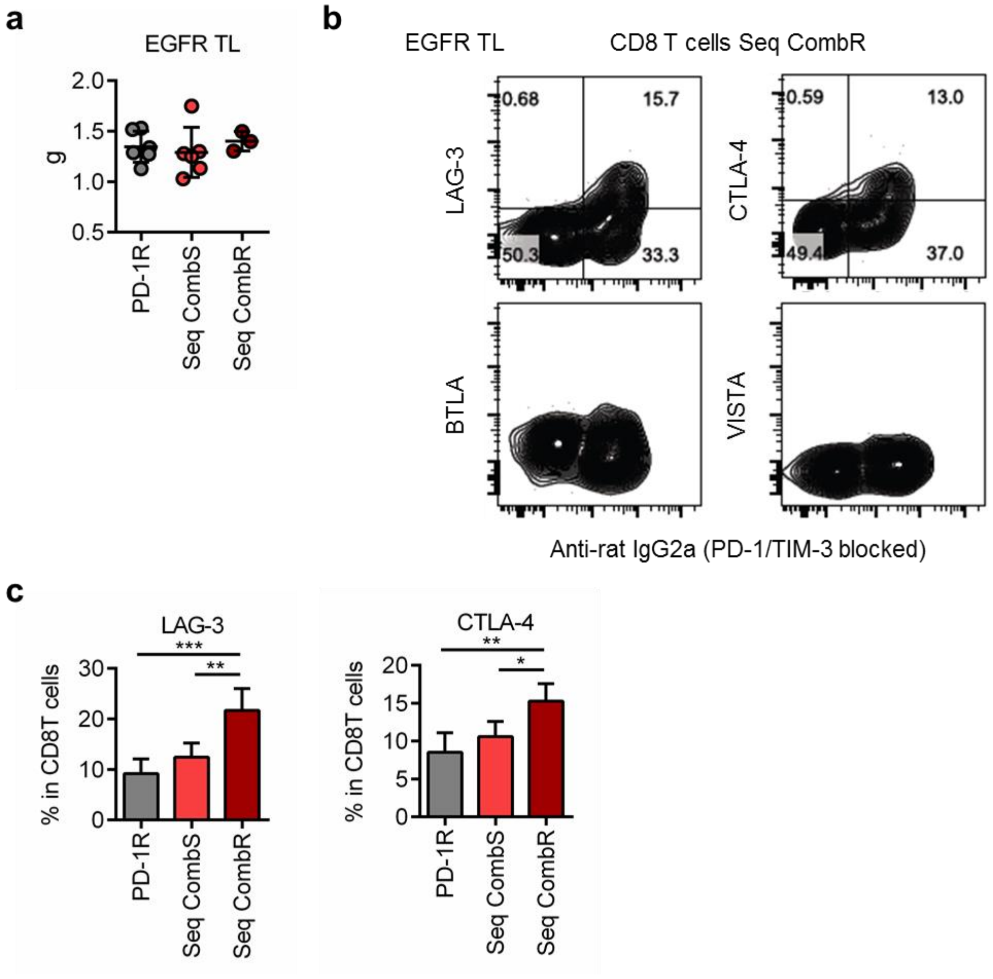

Supplementary Figure 5. Anti-tumor efficacy and characteristics of T cells from EGFR TL mice treated with PD-1 and TIM-3 sequential combination

(a) Total lung weight of PD-1 resistant (PD-1R) (n=6), sequential anti-TIM-3 treated EGFR TL mice following anti-PD-1 blockade failure (Seq combS: n=6) and sequential combination resistance (Seq combR; n=3). (b) Representative flow cytometry data showing CD8 T cells from sequential combo resistant tumors (Seq combR) with both PD-1 and TIM-3 therapeutic antibodies bound on their surface express high LAG-3 and CTLA-4 but not BTLA nor VISTA. (c) The time-dependent increase in LAG-3 and CTLA-4 expression in CD8 T cells from sequential combo resistance (Seq combR: n=3) as compared to CD8 T cells from PD-1 resistant tumors (PD-1R) (n=6) and sequential combo sensitive (Seq combS; n=6). Data are shown as mean ± standard deviation. (*P<0.05, **P<0.01. ***P<0.001, one-way ANOVA with Tukey's multiple comparison test)

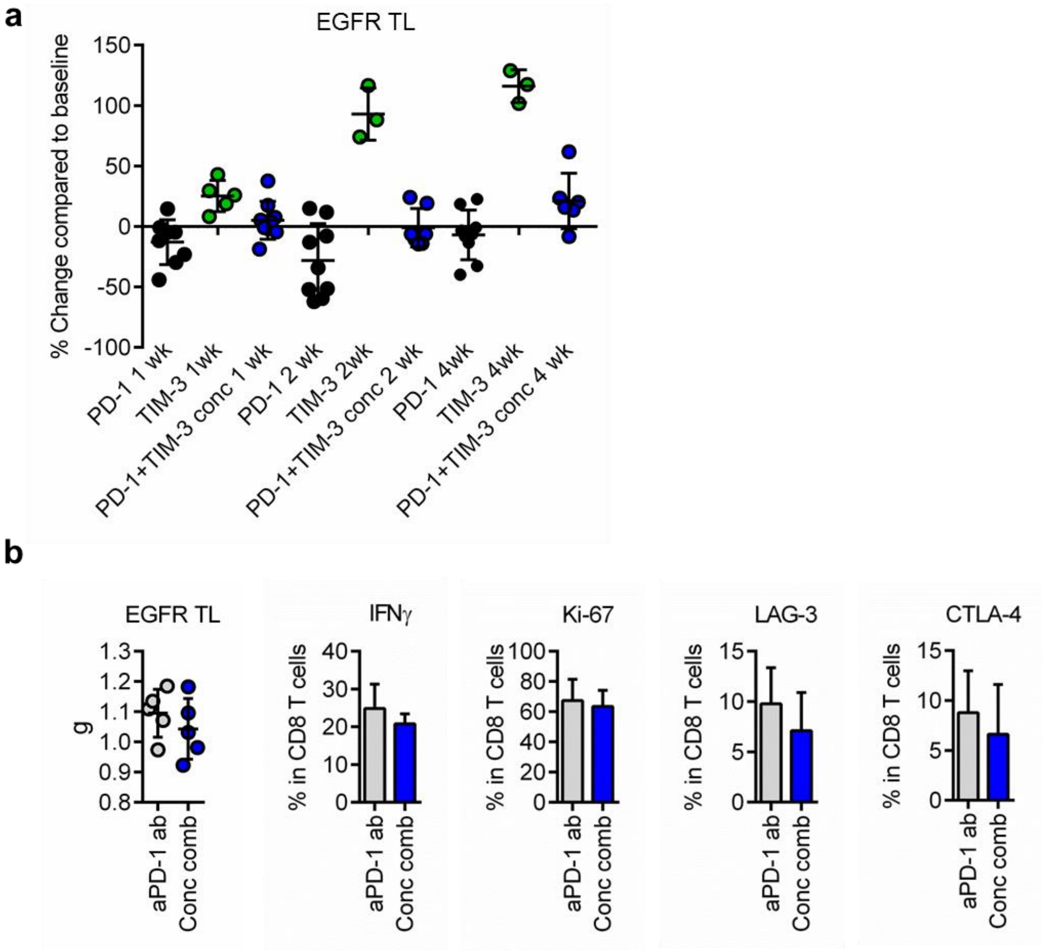

Supplementary Figure 6. Concurrent PD-1 and TIM-3 blockade did not show any significant advantage in anti-tumor effect in EGFR TL mice

(a) Comparison of MRI tumor volume measurement of PD-1 antibody treatment alone or PD-1 and TIM-3 antibody concurrent treatment. Each data point represents tumor volume of a different mouse at that time point. **(b)** Total lung weight and lung tumor immune analysis including T cell activation and checkpoint markers in short term PD-1 (n=5) or PD-1 and TIM-3 antibody treated mice (concurrent comb) (n=5). Data are shown as mean ± standard deviation.

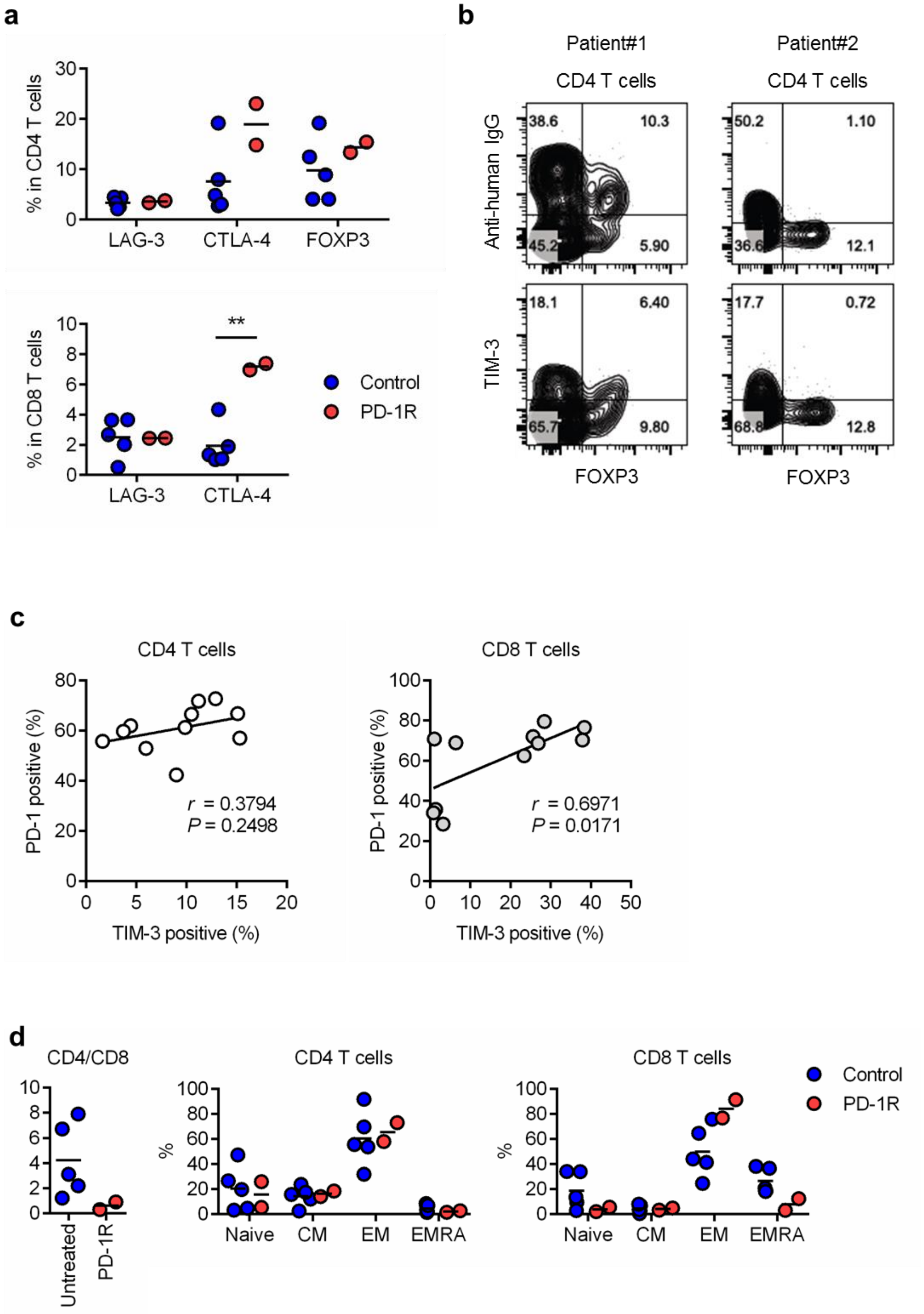

a
% in CD4 T cells
30
20
10
0
LAG-3
CTLA-4
FOXP3
% in CD8 T cells
10
8
6
4
2
0
**
LAG-3
CTLA-4
Control
PD-1R
b
Patient#1
CD4 T cells
Patient#2
CD4 T cells
Anti-human IgG
38.6
10.3
45.2
5.90
50.2
1.10
36.6
12.1
TIM-3
18.1
6.40
65.7
9.80
17.7
0.72
68.8
12.8
FOXP3
FOXP3
c
CD4 T cells
PD-1 positive (%)
r = 0.3794
P = 0.2498
TIM-3 positive (%)
CD8 T cells
PD-1 positive (%)
r = 0.6971
P = 0.0171
TIM-3 positive (%)
d
CD4/CD8
Untreated
PD-1R
CD4 T cells
%
Naive
CM
EM
EMRA
CD8 T cells
%
Naive
CM
EM
EMRA
Control
PD-1R

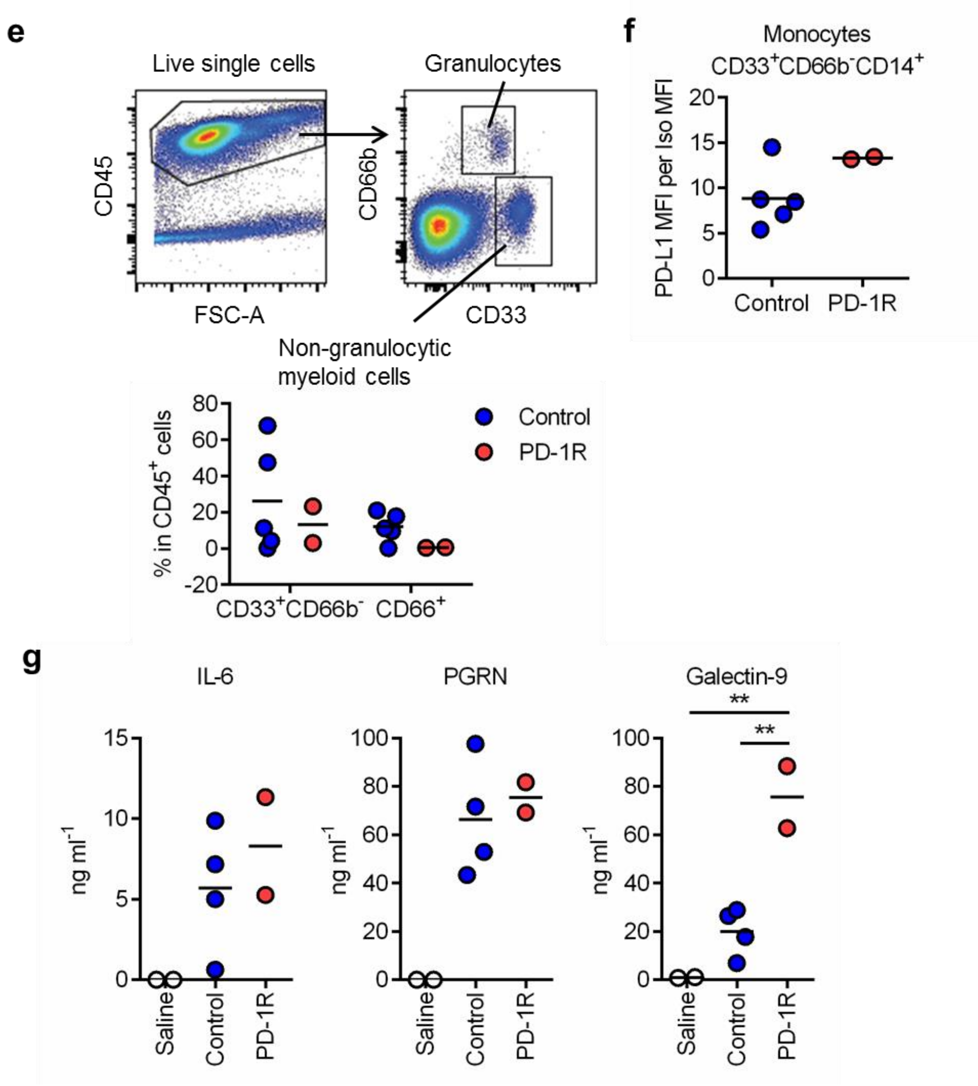

Supplementary Figure 7. Characteristics of immune cells from patient samples

(a) Inhibitory T cell markers in CD4 and CD8 T cells from human effusion samples.

Expression of LAG-3, CTLA-4, and FOXP3 was compared between control effusions from untreated patients (n=5) and two effusion samples from patients whose tumor developed resistance to anti-PD-1 treatment (PD-1R). **P=0.0041, Student's t test. (b) Therapeutic anti-PD-1 antibody binding and TIM-3 expression in regulatory T cells. In the effusion sample from Patient #1, 63.5% or 39.5% of $FOXP3^{+}$CD4 T cells show therapeutic antibody binding and TIM-3 expression. In the effusion sample from Patient #2, less than 10% of $FOXP3^{+}$CD4 T cells show anti-PD-1 antibody binding and TIM-3 positivity. (c) Correlation between PD-1 and TIM-3 expression in CD4 and CD8 T cells from surgically resected tumor samples. The expression of PD-1 and TIM-3 was evaluated in CD4 and CD8 T cells from surgically resected non-small cell lung tumor tissues (n=11). A positive correlation between PD-1 and TIM-3 was detected in CD8 T cells but not CD4 T cells. (d) Characteristics of T cells in

patient effusion samples. Left: CD4/CD8 ratio in anti-PD-1 resistant samples (PD-1R) compared to control. Mean of CD4/CD8 ratio in effusions: Control (Con) vs anti-PD-1 resistant (PD-1R) =4.231 vs 0.605 (P=0.1594). Right: ratio of each T cell subset in CD4 and CD8 T cells in effusions from two PD-1R patients compared to control (n=5). T cells were classified into naive: $CD45RA^{+}CCR7^{+}$, central memory (CM): $CCR7^{+}CD45RA^{-}$, effector memory (EM): $CCR7^{-}CD45RA^{-}$, effector memory re-expressing RA (EMRA): $CCR7^{-}$ $CD45RA^{+}$. Mean of EM CD8 T cells: Control vs PD-1R = 50.12% vs 84.10%. (e,f) Characteristics of myeloid cells in patient effusion samples. No significant change was detected in major myeloid cell populations; granulocytes ($CD66b^{+}$) and non-granulocytic myeloid cells ($CD33^{+}CD66b^{-}$) between untreated vs PD-1R samples (e). PD-L1 expression was evaluated in monocytes ($CD33^{+}$ $CD66b^{-}CD14^{+}$ cells). Mean of fold increase in PD-L1 MFI (effusion: untreated vs PD-1R=8.849 vs 13.33 (f). (g) IL-6, PGRN and Galectin-9 concentrations in supernatants from effusion samples (Saline vs PD-1R; **P=0.0027 and Control vs PD-1R: **P=0.0052, one-way ANOVA with Tukey's multiple comparison test).
Each data point represents a different sample and line represents the mean.

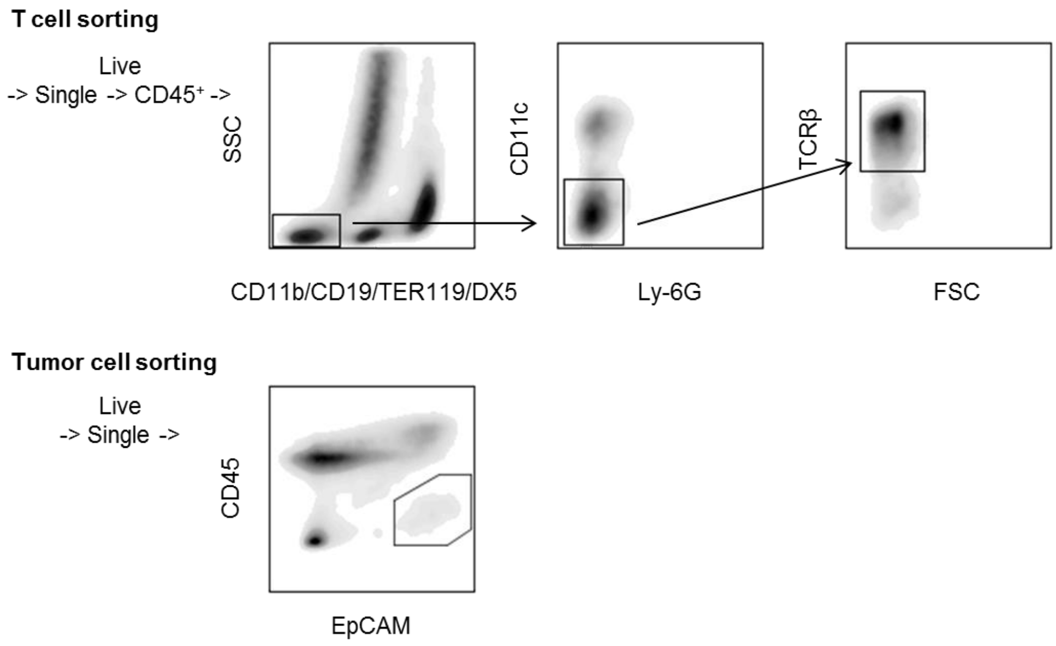

Supplementary Figure 8. Gating strategy for Epcam and T cell sorting by FACS

$CD45^+CD11b^-$ $CD19^-$ $TER119^-$ $DX5^-$ $CD11c^-$ LY-$6G^-$ $TCRbeta^+$ cells were sorted as T cells and $CD45^-Epcam^+$ cells were sorted as tumor cells and used for RNA extraction and sequencing.

Supplementary Table 1: Antibody list for human

Antigen	Clone	Antigen	Clone
CD45	2D1	CCR7	150503
CD3	UCHT1	CD45RA	HI100
CD4	RPA-T4	PD-1	EH12.1
CD8	RPA-T8	TIM-3	F38-2E2
CD66b	G10F5	LAG3	FAB2319*
CD33	WM53	PD-L1	29E.2A3
CD14	M5E2	FOXP3	234A/E7
CD45RO	UCHL1	CTLA-4	14D3

*Catalog number (R and D systems)

Supplementary Table 2: Antibody list for mouse

Antigen	Clone	Antigen	Clone
CD45	30-F11	CD44	IM7
CD3ε	145-2C11	PD-1	29F.1A12
CD4	RM4-5	TIM-3	RMT3-23
CD8a	53-6.7	LAG-3	C9B7W
CD19	6D5	BTLA	8F4
DX5	DX5	VISTA	MH5A
NKp46	29A1.4	PD-L1	10F.9G2
CD11c	N418	CEACAM1	CC1
CD11b	M1/70	FOXP3	FJK-16s
Ly6G	1A8	CTLA-4	UC10-4B9
CD103	2E7	IFNγ	XMG1.2
TCRβ	H57-597	Ki-67	16A8
TER-119	TER-119	Galectin-9	RG9-35

Supplementary Methods:

Mouse lung tumor and immune cell characterization

Mice were sacrificed, and blood was collected through cardiac puncture; lungs were then perfused with cold PBS containing 5 mM EDTA from the right ventricle after collecting BAL fluid. Whole normal or tumor bearing lung was resected and one left lobe and five right lobes were used for histological and flow cytometry analysis. Lung lobes were shredded into small pieces and incubated in collagenase containing buffer: 100 units/ml of collagenase type IV (Invitrogen), 10 μg/ml of DNase I (Roche), and 10% FBS in RPMI1640 medium for 45 min. After incubation, cells were treated with RBC lysis buffer and passed through cell strainer to remove debris. The cell pellet was dissolved by 2% FCS in HBSS, stained with antibodies at 1:50 dilution and used for flow cytometry analysis.
